# Circuits regulating pleasure and happiness: the evolution of reward-seeking and misery-fleeing behavioral mechanisms in vertebrates

**DOI:** 10.3389/fnins.2015.00394

**Published:** 2015-10-23

**Authors:** Anton J. M. Loonen, Svetlana A. Ivanova

**Affiliations:** ^1^Department of Pharmacy, Geestelijke GezondheidsZorg Westelijk Noord-Brabant Chair of Pharmacotherapy in Psychiatric Patients, University of GroningenGroningen, Netherlands; ^2^Mental Health Institute Westelijk Noord-BrabantHalsteren, Netherlands; ^3^Molecular Biology and Biological Psychiatry, Mental Health Research InstituteTomsk, Russia; ^4^Department of Ecology and Basic Safety, National Research Tomsk Polytechnic UniversityTomsk, Russia

**Keywords:** evolution of CNS, striatum, amygdala, habenula, addiction, depression, ketamine

## Abstract

The very first free-moving animals in the oceans over 540 million years ago must have been able to obtain food, territory, and shelter, as well as reproduce. Therefore, they would have needed regulatory mechanisms to induce movements enabling achievement of these prerequisites for survival. It can be useful to consider these mechanisms in primitive chordates, which represent our earliest ancestors, to develop hypotheses addressing how these essential parts of human behavior are regulated and relate to more sophisticated behavioral manifestations such as mood. An animal comparable to lampreys was the earliest known vertebrate with a modern forebrain consisting of old and new cortical parts. Lampreys have a separate dorsal pallium, the forerunner of the most recently developed part of the cerebral cortex. In addition, the lamprey extrapyramidal system (EPS), which regulates movement, is modern. However, in lampreys and their putative forerunners, the hagfishes, the striatum, which is the input part of this EPS, probably corresponds to the human centromedial amygdala, which in higher vertebrates is part of a system mediating fear and anxiety. Both animals have well-developed nuclear habenulae, which are involved in several critical behaviors; in lampreys this system regulates the reward system that reinforces appetitive-seeking behavior or the avoidance system that reinforces flight behavior resulting from negative inputs. Lampreys also have a distinct glutamatergic nucleus, the so-called habenula-projection globus pallidus, which receives input from glutamatergic and GABAergic signals and gives output to the lateral habenula. Via this route, this nucleus influences midbrain monoaminergic nuclei and regulates the food acquisition system. These various structures involved in motor regulation in the lampreys may be conserved in humans and include two complementary mechanisms for reward reinforcement and avoidance behaviors. The first system is associated with experiencing pleasure and the second with happiness. The activities of these mechanisms are regulated by a tract running via the habenula to the upper brainstem. Identifying the human correlate of the lamprey habenula-projecting globus pallidus may help in elucidating the mechanism of the antidepressant effects of glutamatergic drugs.

## Introduction

Recently, as a way to develop new treatments, we have proposed that neuroscientists should develop new hypotheses to explain how psychic disorders are generated (Loonen, 2014). One of these hypotheses might be that the complexity of depressive mood disorders can be disentangled by postulating the existence of two different, but mutually interacting, neuronal circuits regulating the intensity of anhedonia (lack of pleasure) and dysphoria (lack of happiness; Loonen and Ivanova, submitted). These circuits are functionally dominated by partly closed limbic (misery motivated, avoidant) and extrapyramidal (reward motivated, reinforced) cortico-striato-thalamo-cortical (CSTC) circuits, which motivate the individual to express the corresponding motor behavior. A basic starting point of this hypothesis is that, in its most essential form, behavior can be considered a basic reaction of organisms to important stimuli from the environment, which later became connected to specific emotions. To survive as an individual and a species, even our oldest ocean-dwelling ancestors living over 540 million years ago (mya) must have been able to react to the environment to feed, evade predators, defend territory, and reproduce. Thus, their primitive nervous systems must have regulated the necessary behaviors and incorporated the most essential structures of all today's freely moving *Animalia*. However, the path from the nervous systems of these primitive organisms to those of modern humans is long; the massive expansion of the forebrain may have partly improved the basic functions, but also likely obscured which parts and connections are primary to these behaviors and their links to our more sophisticated constructs related to mood.

For regulating behaviors related to foraging, reproducing, or predator avoidance, the candidate primary structures will be those that regulate movement. Among these structures are the extrapyramidal system (EPS) and the amygdala, which are involved in both motor and reward-related or avoidance pathways. In this article, we analyze the vertebrate origins of the EPS and the amygdala to identify the most essential parts of the circuits that regulate emotions such as pleasure and happiness, tracing them in the vertebrate family tree to their origins in motor-related avoidant and reward circuits. We suggest that taking these connections as a starting point during neuroimaging experiments with pharmacological challenge will lead to a better understanding of the neurobiology of mood and anxiety disorders.

## History of the central nervous system^1^

**Figure 1:**
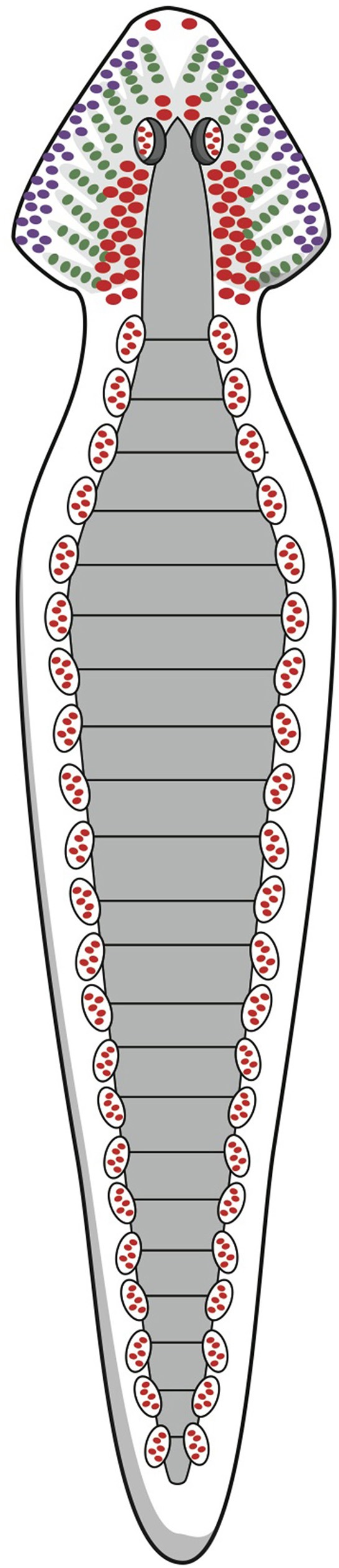
**Schematic representation of the nervous system of the planarian flatworm**. © A. J. M. Loonen.

During the Cambrian explosion, beginning around 542 mya, the first representatives of the animal kingdom arose, although the earliest organisms in this kingdom may date back even farther. These early ancestors included a group of bilateria: animals with bilateral symmetry, i.e., having a front and a back end, as well as an upside and downside, and therefore a left and a right side. Some of these bilateral animals ultimately developed stepwise into vertebrates, including humans. What these primitive forerunners looked like is difficult to estimate. Freshwater planarians are believed to belong to an early arising group of organisms with defined bilateral symmetry, dorsoventral polarity, a central nervous system (CNS), and a simple brain structure (Figure 1; Umesono and Agata, 2009). They are considered to be a model for our earliest ancestors but belong to another branch of the animal kingdom, the phylum Platyhelminthes, rather than the superphylum including the vertebrates (Dunn et al., 2008; Umesono and Agata, 2009). Still, the planarian's brain is how many paleontologists and zoologists would suggest that the last common ancestor of the bilaterian clade must have looked (Northcutt, 2012). Many developmental biologists, however, relying on the roles of genes and gene networks, suggest a completely different last common bilaterian ancestor, arguing that it would have been a morphologically more complex organism, already with a tripartite brain consisting of forebrain, midbrain, and hindbrain (Reichert, 2005; Northcutt, 2012).

### The presence of a notochord (chorda dorsalis)

One of the first steps of the bilaterians on the evolutionary pathway to the human species was the development of a notochord. The notochord is a flexible rod-shaped body found in the embryos of all chordates. Its cells are derived from the mesoderm and the structure defines the axis of the embryo. Among today's animals, the notochord persists post-embryonically in the lancelet. Cephalization followed on the advent of the notochord. A modern representative of these earliest craniates is the hagfish, a jawless fish with a head but no distinct vertebrae. Their closest living relatives, the lampreys, possess both and may be modern-day representatives of the first true vertebrates (Nieuwenhuys, 2002). From these primitive vertebrates, the cartilaginous fishes (sharks and rays) and the bony fishes developed. Today, the bony fishes clade known as the lobe-finned fishes includes the tetrapods, which invaded the continents and gave rise to amphibians, reptiles and birds, and mammals.

The earliest chordates probably already had an axial notochord, flanked by a single, dorsally situated tubular CNS. However, the CNS of these early chordates, now represented by the lancelet, lacks a few essential parts of the craniate brain (pallium, epithalamus, and cerebellum; Nieuwenhuys, 2002). Thus, the first spinal cord might have developed even as the vertebrate brain as we know it today was still in progress.

### Evolution of the brain

To describe how the brain developed in this ancient period, a comparison of the brains of lancelets (amphioxus; Lacalli, 2008), hagfishes (myxine or eptatretus), lampreys (petromyzontidae), and bony fishes (zebrafish *Danio rerio* and ray-finned fishes) may offer a suitable model (Table 1; Murakami et al., 2005). Lancelets lack a defined head, hagfishes lack vertebrae, and lampreys lack a jaw.

**Table 1:**
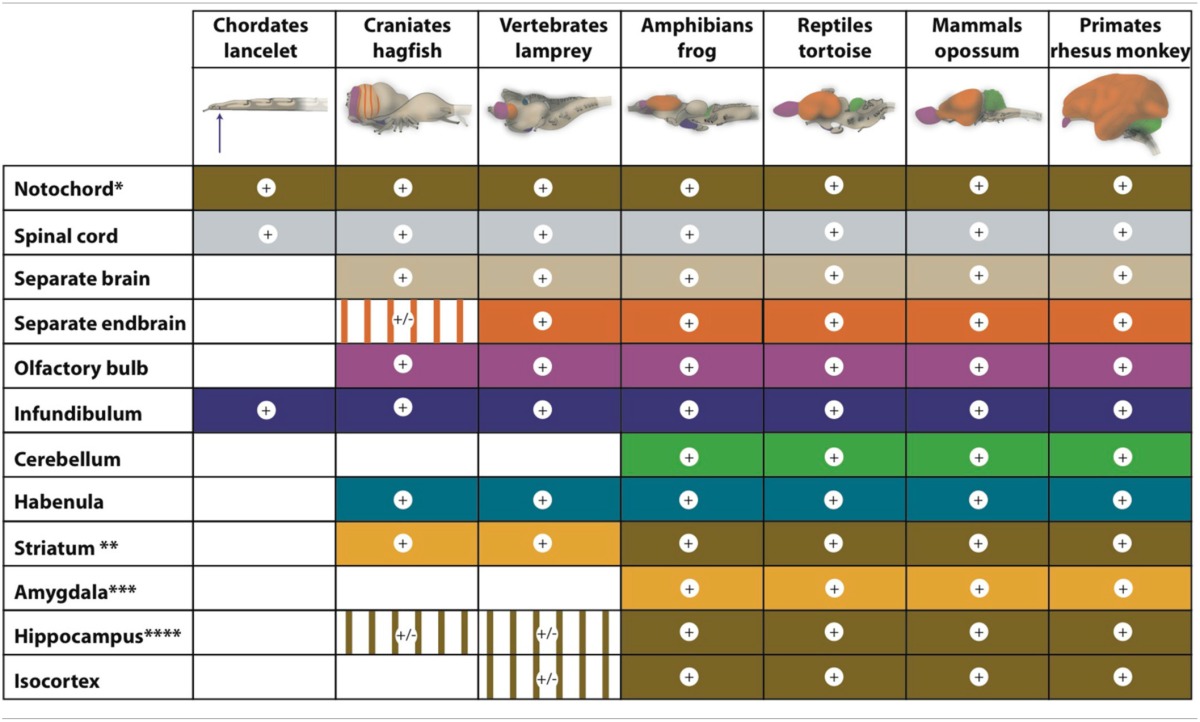
**Development of the central nervous system of possible forerunners of humans**.

^*^Chorda dorsalis (not shown).

^**^In the hagfish and lamprey, the striatum can also be considered a forerunner of the nuclear amygdala.

^***^Only nuclear amygdala is considered; the cortical areas are represented by part of the pallium.

^****^Hippocampal pallium is present in hagfishes and lampreys but not yet clearly developed.

*Figures are modified from Nieuwenhuys (1998)*.

*Color code correspond to structures in figures*.

The cerebellum is the newest brain component. Although lampreys have a structure that is comparable to the cerebellum, their homolog lacks the typical components and connections necessary for cerebellar function. A true cerebellar structure first appeared in amphibians (Murakami et al., 2005).

Lancelets also have a very limited forebrain (Nieuwenhuys, 1998; Murakami et al., 2005). Indeed, morphologically, the lancelet brain is a simple neural tube with no overt segmental compartments; however, its forebrain contains a hypothalamus-like structure (infundibulum; Table 1) that is associated with a ventrally located Hatschek's pit. This last structure is a hypothetical homolog of the pituitary gland (hypophysis; Murakami et al., 2005). In addition, the brainstem contains a locomotor control center roughly comparable to the tegmental and reticulospinal system of more complex chordates (Lacalli, 2008).

Within the hagfish brain, the hindbrain (rhombencephalon), midbrain (mesencephalon), extensive thalamus (diencephalon), and endbrain (telencephalon) can be readily delineated at the dorsal side. Within the endbrain, two evaginated olfactory bulbs and two hemispheres can be distinguished, but the latter remain for a large part fused within the midline (Wicht and Nieuwenhuys, 1998; Nieuwenhuys, 2002). The ventricular system of the hagfish forebrain is small and lacks a lumen in most areas (Jansen, 1930; Wicht and Northcutt, 1993). Moreover, the actual pallium, the forerunner of cortical tissue, seems to be relatively small and surrounds the large central prosencephalic nucleus, which is really a diencephalic structure reaching forward to immediately caudal of the olfactory bulb (Wicht and Northcutt, 1992, 1993). Thus, the rostral (nose) pole of the extensive thalamus appears to be surrounded by a telencephalic mantle, without the latter being a clearly separate structure (shown striped in the figure of the hagfish brain in Table 1). Wicht and Northcutt (1998) have studied the connections of the hagfish pallium and give several reasons for why this pallium is only a homolog of one or two cortical areas of higher developed craniates. Specifically, a dorsal pallium (forerunner of the mammalian isocortex) is still missing. The hagfish cortex is entirely dominated by secondary olfactory projections. Somatosensory and visual afferents relay within the tectum (and tegmentum) and reach the hagfish cortex (pallium) via the diencephalon (extensive thalamus). Descending cortical (pallial) efferents reach the preoptic region, the dorsal thalamus, and the mesencephalic tectum but not the motor and premotor areas of the brainstem.

The lamprey forebrain (prosencephalon) is more developed than that of the hagfish. The lamprey has pallial structures that are comparable to higher vertebrates, but the cerebral cortex and hippocampus, are not yet clearly developed (Nieuwenhuys and Nicholson, 1998; Murakami et al., 2005). In addition, the lamprey has a small dorsal thalamus (the part forming the proper so-called “thalamus” in humans). However, the epithalamus (consisting of the habenula and epiphysis) is well developed in both hagfishes and lampreys (Jansen, 1930).

Similar to the forebrain of lampreys, the endbrain of the zebrafish can be divided into the pallium and subpallium, but the subpallium in these fish consists of two parts: the lateral and medial ganglionic eminences (Panula et al., 2010). Moreover, the pallium has clearly developed medial, dorsolateral, and ventral parts (Murakami et al., 2005) that correspond to a hippocampal pallium, general pallium, and piriform pallium (Nieuwenhuys, 2009). This pattern is roughly comparable to that of an embryonic stage of the human brain.

### The striatum of lampreys and hagfishes

**Figure 2:**
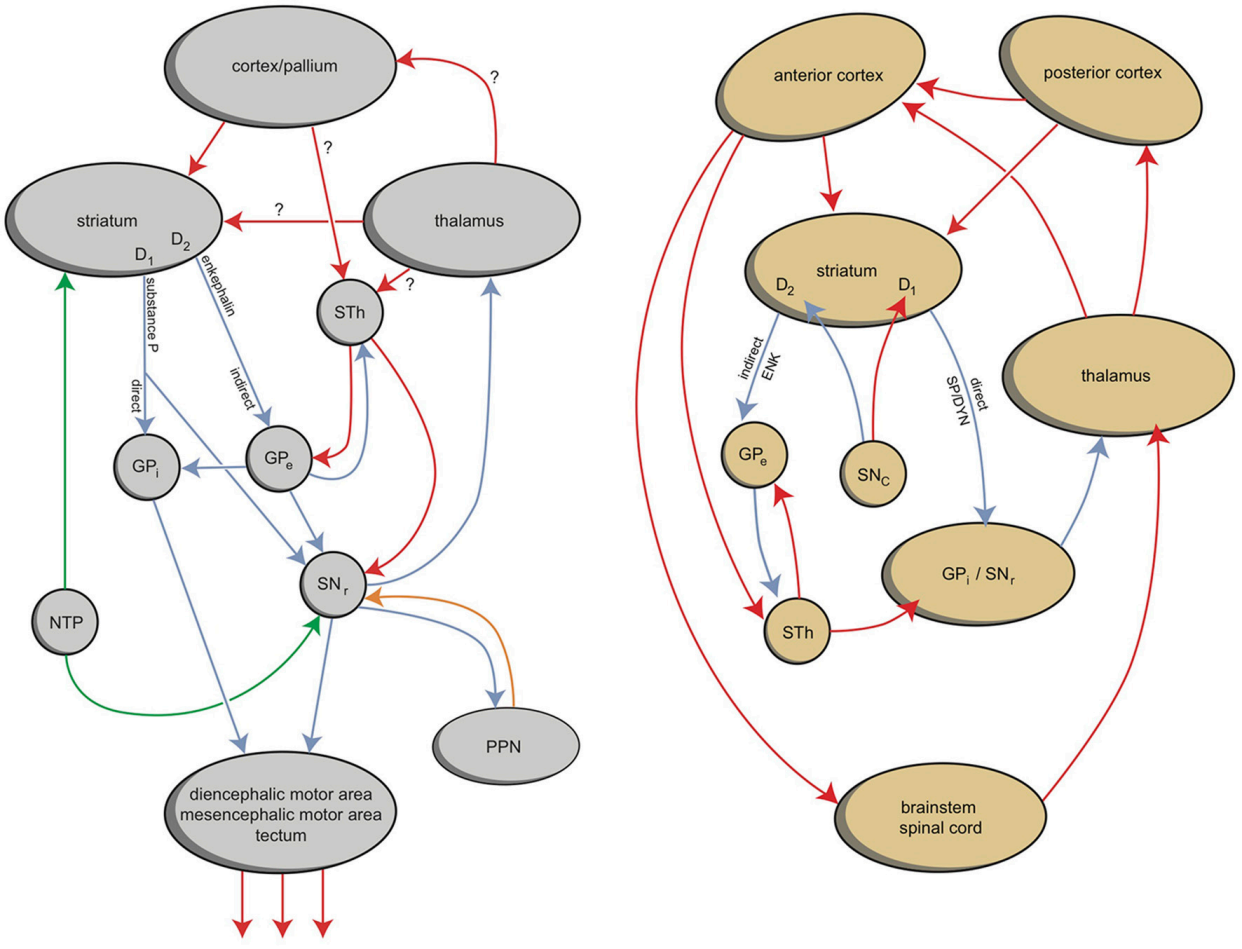
**Simplified representation of the extrapyramidal system of lampreys (left) and humans (right) (Stephenson-Jones et al., 2011, 2012a; Loonen and Ivanova, 2013)**. In lampreys, the internal and external parts of the globus pallidus are intermingled within the dorsal pallidum but functionally segregated. GPe, globus pallidus externa; GPi, globus pallidus interna; NTP, nucleus tuberculi posterior; PPN, pedunculopontine nucleus; SNr, substantia nigra pars reticulata; STh, subthalamic nucleus. Left figure: red, glutamatergic; blue, GABAergic; green, dopaminergic; orange, cholinergic. Right figure: red, excitatory; blue, inhibitory. © A. J. M. Loonen.

According to the work of Sten Grillner and collaborators, adult lampreys have an EPS that is comparable to the homologous system in humans (Robertson et al., 2014). As in all vertebrates, the networks coordinating locomotor movements of lampreys are located in the spinal cord. These so-called central pattern generators coordinate the activity of the different muscles taking part in specific movements. In lampreys, the activity of the central pattern generators is initiated and coordinated from specific command centers in the lower brain, e.g., the diencephalic and mesencephalic locomotor regions (Figure 2). This lower brain-spinal cord locomotor system seems to be under direct control of quite a modern EPS (Figure 2; Loonen and Ivanova, 2013; Robertson et al., 2014). However, an important difference between lampreys and mammals is that in lampreys, the basal ganglia output structures send their commands mainly to the brainstem; in mammals, the commands are sent mainly via the dorsal thalamus to the cerebral cortex, constituting a cortical-ganglio-thalamo-cortical circuit (Figure 2; Loonen and Ivanova, 2013; Robertson et al., 2014). The position of these structures is also slightly different. The lamprey homolog of the dopaminergic substantia nigra/ventral tegmental area (SNc/VTA) is considered to be the nucleus of the tuberculum posterior (NTP), which is localized within the caudal extensive thalamus (Pérez-Fernández et al., 2014). In addition, in the lamprey, the striatum is located caudal to the septal area in the medial wall of the endbrain and thus also near the border of the extensive thalamus (Pérez-Fernández et al., 2014).

Similar to the situation in mammals, dopaminergic fibers of the NTP regulate the activity of the striatum. These dopaminergic fibers are under the control of a stimulatory direct projection from the lateral habenula (epithalamus; Robertson et al., 2014). In addition, an indirect inhibitory pathway runs from the lateral habenula via a GABAergic rostromedial tegmental nucleus (RMTg). These structures increase activity during reward situations and decrease activity when an expected reward does not occur. Lampreys also have a separate glutamatergic nucleus (the habenula-projecting globus pallidus; GPh), which activates the habenula (Robertson et al., 2014). This nucleus receives inhibitory control from the striatum and excitatory input from both the thalamus and pallium. Thus, the activity of the dopaminergic NTP is under the control of an evaluative system with input from the striatum and pallium for determining whether the locomotor activity results in reward or not (Robertson et al., 2014). Cholinergic circuitry from the medial habenula to the interpeduncular nucleus and periaqueductal gray regulates the fear/flight response (Robertson et al., 2014).

Whether hagfishes have an EPS comparable to that of lampreys is uncertain. We have found no evidence in the literature for such a system by mapping dopamine receptors. Indeed, the catecholaminergic system is much more restricted than in most other craniates, and cell bodies containing dopamine seem to be constrained to the infundibular hypothalamus (Wicht and Nieuwenhuys, 1998). Hagfishes have a striatum, however, that flanks the central prosencephalic nucleus. In the hagfish, all cortical (pallial) fields and the septum receive strong (secondary) input coming from the olfactory bulbs and a moderate secondary olfactory input to the striatum (Wicht and Northcutt, 1993). Nevertheless, the striatum is hardly the destination of cortical efferents. Fibers from the striatum run to the ventral thalamus (hypothalamus) and to the midbrain (tectum, superior raphe, and a possible homolog of the locus coeruleus) (Wicht and Northcutt, 1998).

### In summary

The development of the forebrain may be considered (quite clearly) to have started with an ancestor comparable to today's hagfish. The behavior of this hypothetical animal was dominated by its olfactory input. Its pallium is the forerunner of the archicortex and palleocortex of mammals and played only a minor role in directly estimating motor behavior. These structures give rise to the hippocampal complex, amygdala, and older limbic cortex in humans. Motor behavior was possibly controlled by the striatum, although it is unclear whether the EPS had already been developed. In phylogenically younger craniates, the influence of olfactory input gradually decreased, and the isocortex started to develop. Initially, this new cortical structure did not directly control movement and other behavioral responses. In organisms comparable to lampreys, this control was probably mainly a function of the preoptic hypothalamic area and the basal ganglia. It is likely that the invasion of land by tetrapods had a big effect on the magnitude of the sensory input through the dorsal thalamus. On the output side, the isocortex, the main part of the cerebral cortex in humans, may have started at this point to influence central (motor) pattern generators directly, and the EPS was only adapting the activity of this motor system. The cerebellum arose parallel to the development of this role of the isocortex and has been an increasingly important structure since the evolution of amphibians.

## But what happened from there?

The forebrain of an animal comparable to lampreys may be proposed as the first modern prosencephalon with old and new components. But how did the evolutionary pathway continue from there? It is tempting to speculate that the cortical and subcortical structures are highly conserved during evolution and have retained their organization and function; however, this scenario is not necessarily the case. Newly added tissues may have acquired their function and applied it in a different way. Moreover, anatomical homologies are often easily assumed but take an enormous effort to be sufficiently proven. Nevertheless, with all restrictions, a few conclusions can be drawn when comparing the forebrain of lampreys and their first successors, the amphibians, with that of humans. We concentrate here on the EPS, the amygdala and the epithalamus.

### Evolution of the CSTC circuits

The lamprey EPS is localized slightly differently from that of mammals (Figure 2; Stephenson-Jones et al., 2012a). The more primitive animal's putative SNc/VTA is situated within the extensive thalamus near its border with the midbrain (Pérez-Fernández et al., 2014). In amphibians, the majority of the dopaminergic neurons are localized within the extensive thalamus (Marín et al., 1998; Smeets et al., 2000) and in younger vertebrates, the majority of the dopaminergic SNc/VTA neurons are localized within the midbrain.

At the nose side in lampreys, the striatum is located in the medial wall of the endbrain, caudal to the septal area and dorsal to the preoptic area (Pérez-Fernández et al., 2014). In amphibians, the most medial and rostral parts of this structure become striatum while the lateral and more caudal parts become amygdala (Moreno and González, 2006). With the lamprey, the isocortex starts to develop. In the hagfish, a possible forerunner is still missing, and the striatum receives input from the olfactory bulbs (Wicht and Northcutt, 1998). Therefore, the hagfish striatum is more likely an ancestor of the human nuclear amygdala than of the human striatum. When the isocortex developed in younger vertebrates and took over controlling locomotion, the extrapyramidal striatum took shape in parallel. It has been suggested that this evolution occurred in a modular fashion: When a new capability was acquired, this acquisition should correspond to an expansion of both the cerebral cortex and basal ganglia (Grillner et al., 2013; Robertson et al., 2014). This parallel development may also have resulted in another organization in which motor output was primarily generated by the cerebral cortex and the basal ganglia modified this output within a CSCT circuit.

**Figure 3:**
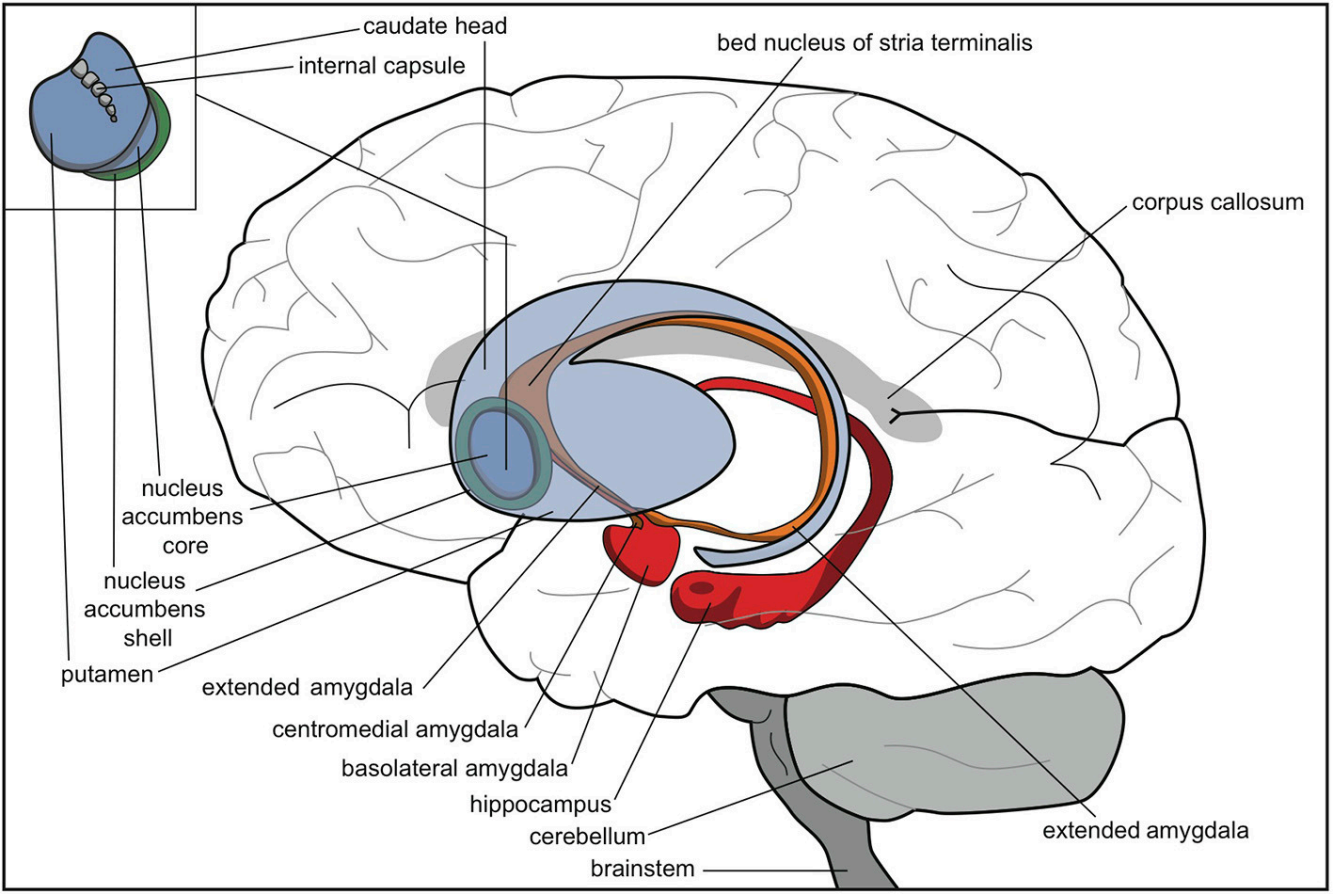
**Human brain with striatum (blue), nuclear amygdala (orange), cortical amygdala (red), and hippocampus (red)**. © A. J. M. Loonen.

Thus, the extrapyramidal regulation of the intensity of motor output possibly developed stepwise whenever the animal acquired a new opportunity to react to its current circumstances (Grillner et al., 2013; Robertson et al., 2014). Topographically, these modules consisted of an expansion of the lateral side of the original striatum, parallel to the development of the isocortex (Figure 3). We suggest that some functions of the lamprey striatum, such as promoting reward-bringing or misery-fleeing behavior, were conserved and extended within the most medial part of this newer striatum, i.e., the accumbens nucleus. Another function of the original lamprey striatum, selecting relevant sensory input for response, may have largely remained within the nuclear amygdala and related nuclear structures.

### Evolution of the amygdaloid complex

**Figure 4:**
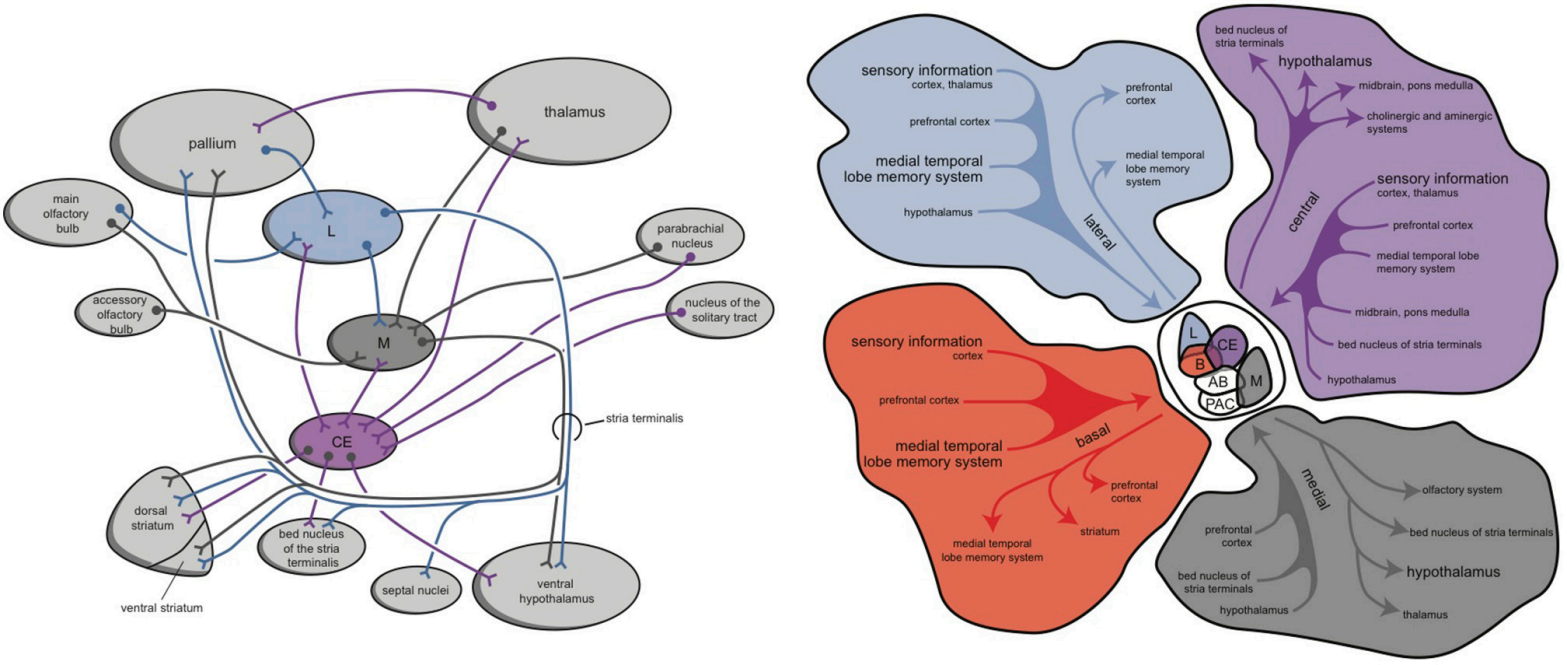
**Simplified representation of the amygdaloid complex of anurans (left) and rats (right) (Pitkänen, 2000; Moreno and González, 2006; right figure reproduced with permission of author)**. CE, central amygdala; L, lateral amygdala; M, medial amygdala. © A. J. M. Loonen.

Within hagfishes, behavioral (motor) output seems to be almost entirely controlled by the preoptic area and striatum. In lampreys, value-based selection of behavior (salience) is largely controlled by the basal ganglia (Robertson et al., 2014). The connectivity of the mammalian amygdala is extremely elaborated and complex (Whalen and Phelps, 2009). This amygdaloid complex consists of 13 nuclei and cortical regions and their subdivisions (Figure 4; Pitkänen, 2000; Whalen and Phelps, 2009). In assuming that the lamprey forebrain is almost entirely incorporated into the later amygdaloid complex, it is interesting to consider the amphibian brain to estimate its relationship with the younger cortex; indeed, in anurans (frogs and toads), the amygdaloid complex is generated embryologically from both cortical (pallial) and subcortical (subpallial) structures. The frog amygdala consists of lateral, anterior, central, and medial components (Figure 4; Moreno and González, 2006). The medial and central amygdala are continuous with the striatum at the front and with dorsal pallidum at the back (Moreno and González, 2006). Considering the connectivity of these structures, the medial amygdala receives and integrates olfactory and vomeronasal information with other sensory information and sends output through the stria terminalis to the hypothalamus. The lateral amygdala receives different sensory information, either by direct or indirect pathways, and the majority of the output is given to the medial and central amygdala. The central amygdala is considered to be the main component of the amygdaloid autonomic system, mediating many autonomic, somatic, endocrine, and behavioral responses (Moreno and González, 2006).

In mammals, the ventral striatum is at a distance continuous with the centromedial amygdala by means of a circular extended amygdala (Heimer, 2003). The bed nucleus of the stria terminalis is the most distant part of the extended amygdala, bordering with the shell part of the nucleus accumbens (Figure 3). In anurans, however, the homolog of the bed nucleus of the stria terminalis appears to have another position (Moreno and González, 2006). Careful analysis, though, has revealed that the anuran nucleus shares many features with its counterparts in more recent tetrapods and is likely to control similar reflexes, responses, and behaviors (Moreno et al., 2012). Future research should reveal the exact evolutionary relationship of this bed nucleus and the rest of the (extended) amygdala.

During evolutionary development from amphibians to mammals, the original connectivity of the amygdala was conserved to a remarkably high degree. The cortical amygdala in mammals is constituted by olfactory and vomeronasal areas while the medial amygdala is considered to be the main vomeronasal secondary relay center. The central amygdaloid component originates long descending pathways (Moreno and González, 2006). The vomeronasal system plays a crucial role in co-specific recognition and reproductive processes in amphibians, reptiles, and mammals, although in humans this system may have lost its importance. The multimodal integration of sensory information is a function of the cortical and basolateral amygdala and results in emotional memory and response generation. The mammalian central nucleus of the amygdala is the main component of the autonomic amygdaloid subdivision. In all mammalians, a long and sole fiber bundle is found, the stria terminalis, interconnecting the amygdala with the rostromedial forebrain (e.g., septal areas and the nucleus of the diagonal band) and hypothalamus. The first branches may (hypothetically) relay to fibers connecting septal areas with the habenula and play an important role in the decision-making process. The second may be crucial to having the hypothalamus initiate the complex emotional response (**?**).

Thus, although several details have not yet been elucidated, many characteristics of the mammalian amygdaloid complex and extended amygdala seem to be directly derived from the lamprey forebrain. This relationship suggests that value-based selection of behavior (salience attribution), which is a function of the lamprey striatum, also is consolidated within the (nuclear) amygdala.

### Evolution of the habenula projection system

The habenula in the epithalamus has recently received much attention for possibly playing a role in depression and addiction (Savitz et al., 2011; Schneider et al., 2013; Belzung et al., 2014; Velasquez et al., 2014; Antolin-Fontes et al., 2015). This hypothesis is strongly related to the influence of the habenula on the activity of monoaminergic control centers of the brainstem (Velasquez et al., 2014; Antolin-Fontes et al., 2015). The habenula is subdivided into two nuclei: the medial habenula and lateral habenula. In lampreys, a direct pathway runs from the homolog of the lateral habenula to the NTP (considered to be a homolog of the SNc/VTA), next to a pathway to a homolog of the inhibitory GABAergic RMTg, which inhibits the NTP (Stephenson-Jones et al., 2012b; Robertson et al., 2014). Other efferents of the lateral habenula run to (diencephalic) histaminergic and serotonergic areas. In lampreys, a projection system from the homolog of the medial habenula to the (cholinergic) interpeduncular nucleus was also identified. These habenular output structures are well conserved across species. All vertebrates examined possess the same efferent pathway, called the fasciculus retroflexus, running to the ventral midbrain (Aizawa et al., 2011; Velasquez et al., 2014; Antolin-Fontes et al., 2015). In mammals, the medial habenula projects almost exclusively to the interpeduncular nucleus (ACh) (Viswanath et al., 2014), whereas the lateral habenula projects to a variety of nuclei including the RMTg (GABA), raphe nuclei (5-HT), SNc (DA), VTA (DA), and the nucleus incertus (GABA) (Aizawa et al., 2011). Moreover, the medial habenula has direct output to the lateral habenula and may regulate the latter's activity (Velasquez et al., 2014; Viswanath et al., 2014).

The input to the epithalamus appears to be less well conserved during evolution, however. In lampreys, the input of the homolog of the medial habenula comes from the medial olfactory bulb, the parapineal organ, the pretectum, and the striatum (Stephenson-Jones et al., 2012b). The input of the lateral habenula comes from the subhippocampal lobe (the GPh) and the lateral hypothalamus, but not from the diagonal band of Broca. Mammals do not have a distinct GPh. It has been suggested that its homolog in primates is localized in the “border of the globus pallidus interna” (GPb) (Robertson et al., 2014; Grillner and Robertson, 2015). Whether the function of the lamprey GPh is retained within this GPb is far from certain.

**Figure 5:**
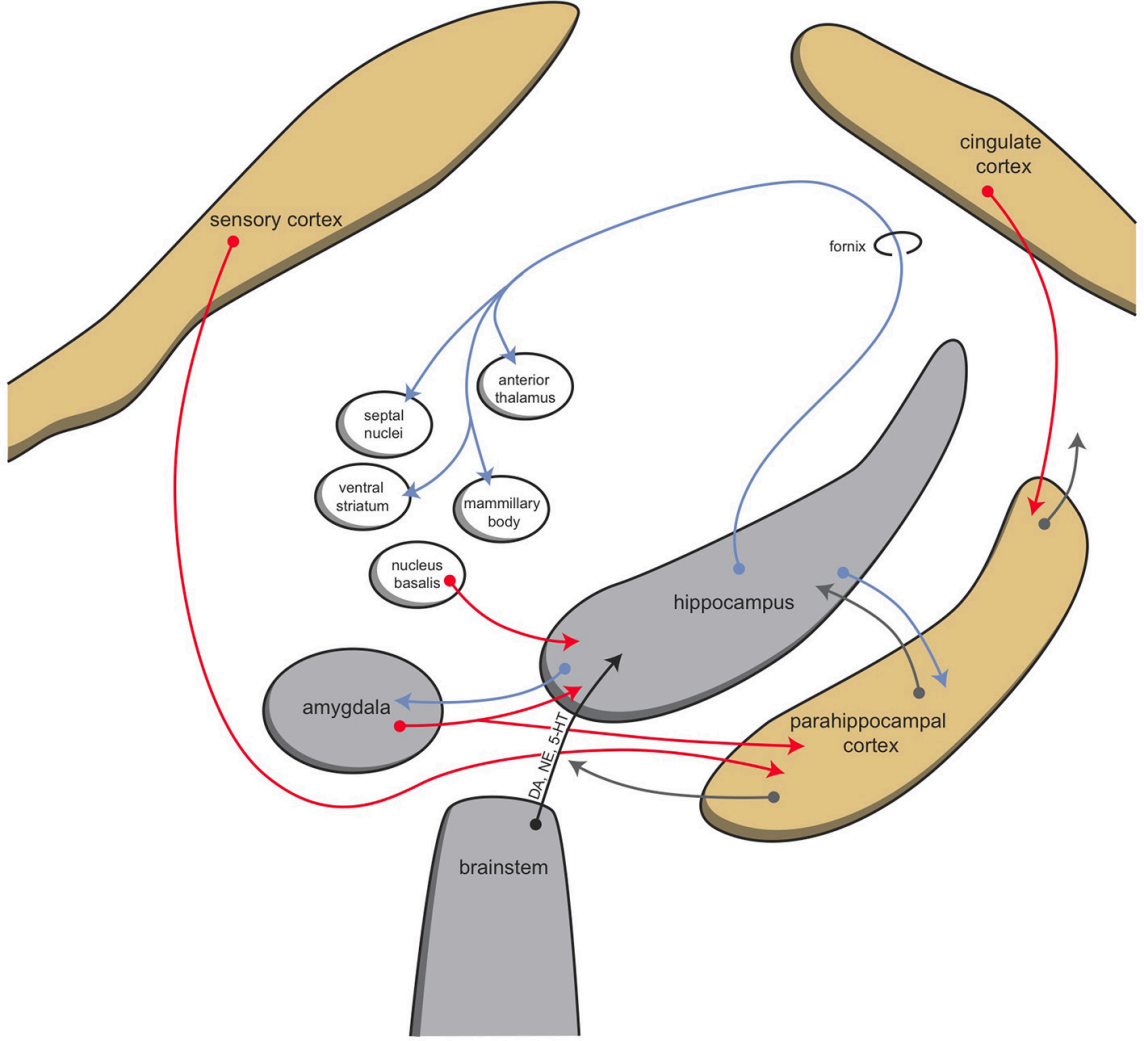
**Schematic representation of connectivity of the hippocampal complex**. © A. J. M. Loonen.

The mammalian habenula receives input via the stria medularis from the posterior septum, as well as from the medial septum, the nucleus of the diagonal band, and midbrain structures (Viswanath et al., 2014; Antolin-Fontes et al., 2015). Major input to the medial habenula arises from the septal nuclei, which in turn receive the majority of their input from the hippocampus (Figure 5; Stephenson-Jones et al., 2012b). Afferents of the lateral habenula come from the hippocampus, ventral pallidum, lateral hypothalamus, globus pallidus, and other basal ganglia structures (Velasquez et al., 2014). It is hypothesized that during evolution from lampreys to mammals, the originally direct sensory innervation of the habenula was replaced by inputs from the so-called limbic system (i.e., the septum and diagonal band of Broca) (Stephenson-Jones et al., 2012b).

Still, it is tempting to speculate that in humans, the amygdala also gives input to the habenula routing via the stria terminalis, septal areas, or lateral hypothalamus and stria medularis. The nuclear amygdala represents the lamprey striatum, which is connected to the lateral habenula via the GPh. However, which structure represents or took over the influence of the lamprey GPh remains to be determined. A strong influence of human dorsal basal ganglia (striatum) on the lateral habenula is also evident, though, and may correspond to a modular expansion of the extrapyramidal circuitry during evolution (Grillner et al., 2013; Robertson et al., 2014).

## Interpretation

Studying and comparing the brains of organisms that may represent earlier stages of human evolution may help us to understand how human emotions are regulated. The ancestor of the so-called isocortex, the newer part of the human cerebral cortex, occurs fairly late in evolution in animals comparable to lampreys. Until then, olfactory sensations appear to have constituted the dominant sensory input, and they regulate output through the preoptic hypothalamic region and the primitive striatum. Sten Grillner's group has developed a detailed model for the regulation of behavioral output in lampreys. They describe an EPS that is largely comparable to the one in humans (Figure 2). However, the lamprey striatum is probably the forerunner of the human nuclear amygdala instead of the human striatum. It may be assumed that a major part of the lamprey forebrain is finally incorporated into the later human amygdaloid complex. It is, therefore, also likely that the human amygdala is still involved in regulating the behaviors controlled by the lamprey forebrain, such as validating the importance of sensory input and selecting the proper behavior for reacting. For this reason, we believe that the human amygdala plays a major role in salience attribution, and controls behaviors like exploring the possibilities of obtaining reward or escaping dangers.

Moreover, the lamprey striatum gives output to motor control centers in the lower brain while the human striatum gives output mainly to the cerebral cortex. However, it can be assumed that the original organization is at least partly retained within humans. The dorsal EPS has an important influence on the brainstem motor centers, which, for example, regulate muscle tonus. The central amygdaloid nucleus provides considerable output to the midbrain, pons and medulla (Pitkänen, 2000) and can be considered also to have maintained this characteristic of the striatum of lampreys. In lampreys, the activity of the behavior-regulating system is adapted by the epithalamus (habenula). This system regulates the activity of the dopaminergic and serotonergic projection systems of the upper brainstem, which also may be true in humans.

**Figure 6:**
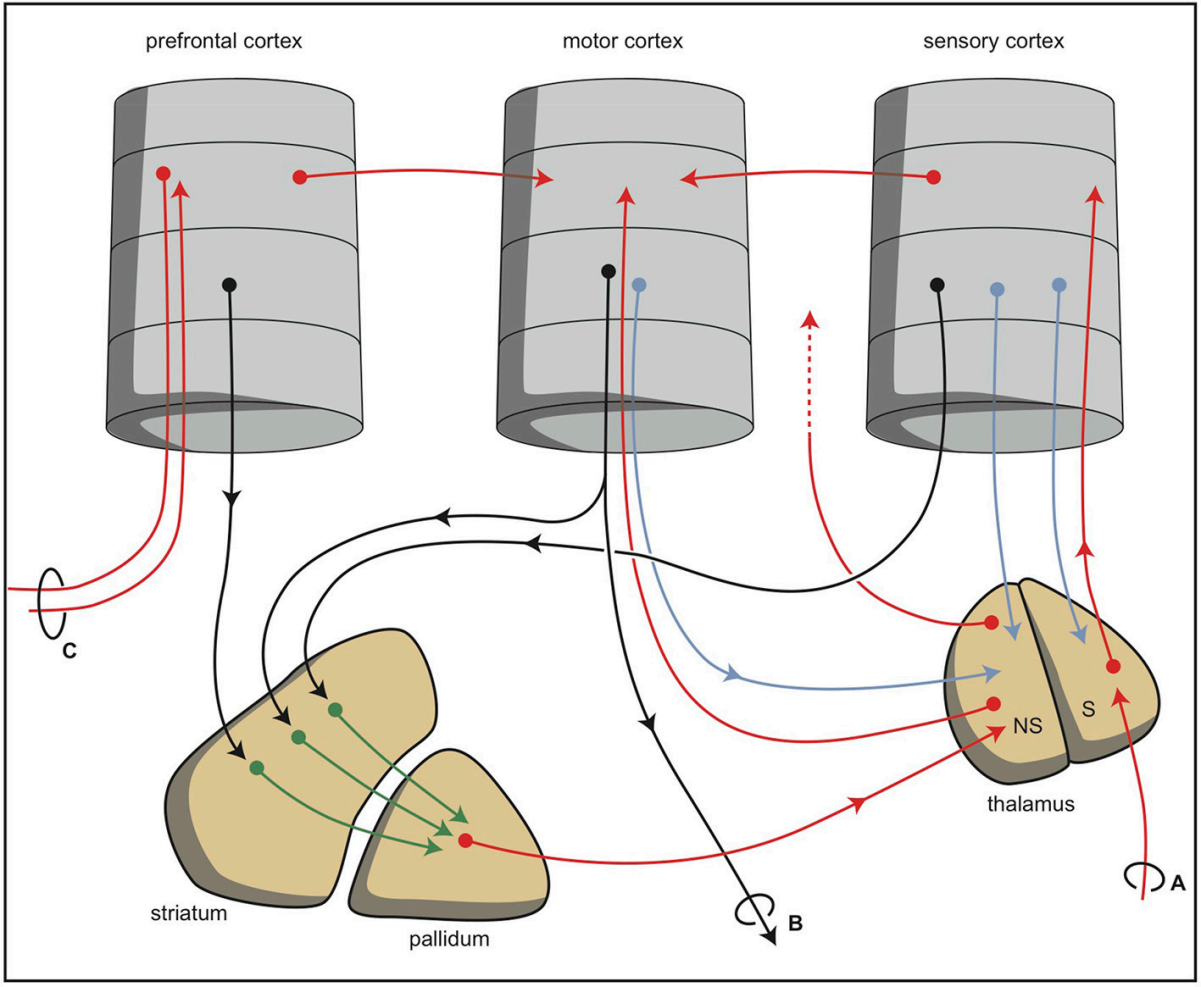
**Simplified representation of the cortico-striatal processing unit in which cortical information leading to a movement is processed via an intra-cortical and (parallel) extra-pyramidal route (Loonen and Ivanova, 2013)**. The converging organization of the extrapyramidal circuits results in a re-entry circuit. **(A)** Sensory input, **(B)** projections to brain stem and spinal cord, and **(C)** projection to and from ipsilateral and contralateral cortical areas. NS, non-specific part; S, specific part. © A. J. M. Loonen.

**Figure 7:**
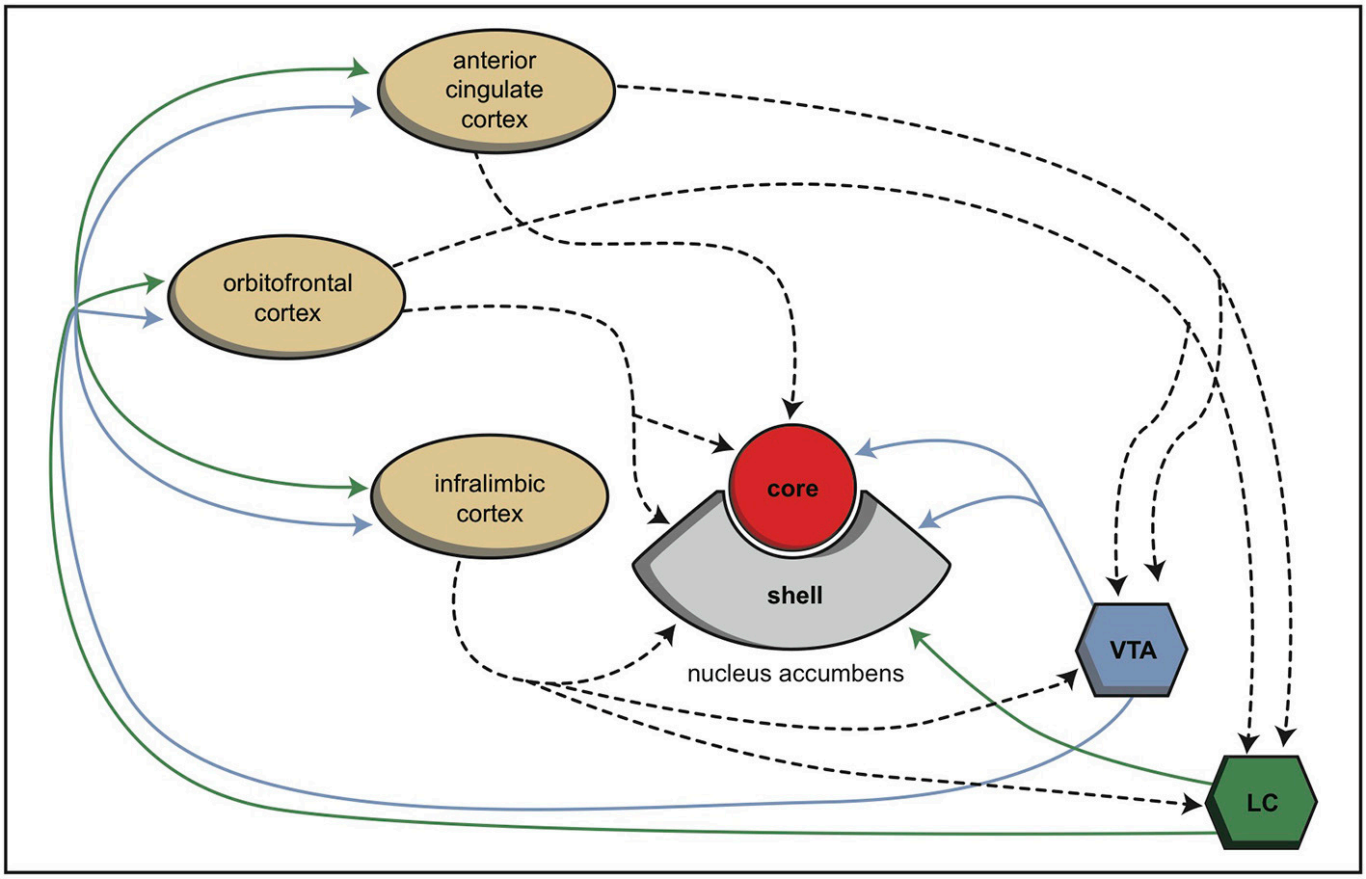
**Schematic representation of the cortical input to both parts of the nucleus accumbens in rats (Dalley et al., 2008)**. © A. J. M. Loonen.

During vertebrate evolution, the EPS has expanded in a modular fashion (Grillner et al., 2013; Robertson et al., 2014). At the dorsal side, this expansion has resulted in circuits involving the putamen and caudate nucleus. At the limbic side, it may have resulted in an extended amygdala and the inclusion of the bed nucleus of the stria terminalis. The accumbens nucleus developed in between these two systems and indeed is considered as the interface between the extrapyramidal and limbic systems (Groenewegen and Trimble, 2007). The converging nature of the cortical input to the extrapyramidal circuits results in several extrapyramidal re-entry circuits starting and ending within the frontal cortex (Figure 6; Loonen and Ivanova, 2013). We have suggested that three of these re-entry circuits involve separate parts of the accumbens nucleus. The core part of the accumbens is the entry station of two re-entry circuits involving the orbitofrontal cortex and the anterior cingulate cortex (Figure 7). The shell part is the entry station for two putative re-entry circuits involving the orbitofrontal cortex and the infralimbic cortex (also known as the subgenual anterior cingulate or Brodmann area 25). This latter area is known to be hyperactive in depression and dysphoria (**?**) and thus may take part in the process of escaping from misery (or, in its originating structure, avoidance behavior in the lamprey).

We hypothesize that two reciprocally active subcortical systems regulate motivation to show reward-gaining or misery-escaping behavior (**?**). These systems are represented by two cortical-ganglial-diencephalic-cortical regulatory systems that contain extrapyramidal (caudate, putamen, and accumbens core) and limbic (centromedial amygdala, extended amygdala, bed nucleus of the stria terminalis, and accumbens shell) basal ganglia as the first input stations. The nucleus accumbens has a central position and regulates the motivation to engage in these two types of life- and species-saving behaviors. The activity of these motivating systems is regulated in turn by dopaminergic, adrenergic, and serotonergic fibers originating in the upper brain stem. Especially, the dopaminergic VTA is important for promoting reward-seeking behavior.

In all vertebrates studied, the activity of these monoaminergic systems is regulated by the habenula. Provided the input of the habenula during the course of evolution has not changed dramatically, the input of the medial habenula comes indirectly from the limbic system via the septal area. Input to the lateral habenula comes from the medial habenula, lateral hypothalamus, and pallidum. In lampreys, the habenula stimulates the dopaminergic system when food is obtained and inhibits the NTP when the behavior is not successful (Robertson et al., 2014). This role is largely maintained in higher vertebrates like monkeys and rats (Matsumoto and Hikosaka, 2007, 2009; Bromberg-Martin et al., 2010). When this effect is extrapolated to addiction, it might very well be possible that the lateral habenula plays an important role in becoming addicted to certain behaviors and in relapse into these behaviors following a period of abstinence. This role is probably similar to that for a lamprey obtaining food, but at a far more sophisticated level.

A similar mechanism may be postulated for misery-escaping behavior. In lampreys, this avoidance behavior involves the medial habenula and the cholinergic interpeduncular nucleus. The interpeduncular nucleus in turn regulates, for example, the activity of the dorsal raphe nucleus (5-HT) and locus coeruleus (NE),^35^ and the latter neurotransmitters are well known to be associated with regulating stress reactions (including depression and anxiety disorders) in humans. Thus, the same parallel we have hypothesized for reward-producing behavior may also be true for misery-escaping behavior: The most ancient part of the human brain deriving from the lamprey forebrain is primarily involved.

## Directions for future pharmacological and neuroimaging research: What happened to the function of GPh?

Keeping the evolutionary pathway in mind, it would be interesting to study the influence of the centromedial amygdala on the activity level of the EPS by adapting this activity through a pathway including the habenular complex. The centromedial amygdala, as far as we know, does not give rise to a strong direct pathway to the lateral habenula (Pitkänen, 2000; Lecca et al., 2014), but these projections nevertheless definitely exist, and indirect pathways may also contribute. The projections from the lateral hypothalamus, globus pallidus, and prefrontal cortex are mainly glutamatergic (Lecca et al., 2014) and can be modulated by the use of glutamatergic drugs, which can serve as probes in specifically designed neuroimaging experiments. As a matter of fact, the noncompetitive N-methyl-D-aspartate antagonist ketamine has already been demonstrated to decrease metabolism in the (right) habenula in a preliminary positron emission tomography study of treatment refractory-depressed patients (Carlson et al., 2013). What remains to be determined is the role of the amygdala in regulating the activity of this dorsal connection of the forebrain to the upper brainstem and whether this connection may explain ketamine's acute antidepressant activity (Loonen, 2014).

In lampreys, reward-bringing behavior is regulated by the glutamatergic GPh, which is influenced by the lamprey striatum. In humans, the lateral habenula receives both GABAergic and glutamatergic input from the globus pallidus and ventral pallidum, both components of the extrapyramidal circuits (Lecca et al., 2014). Thus, in humans, reward-producing behavior is possibly also regulated by the EPS via a pathway including the (lateral) habenula. In our opinion, the neurochemical background is particularly interesting to investigate: If the characteristics of the GPh are retained in the pallidum of humans, this connection should be glutamatergic. Then, it becomes interesting to study where the input of these pallidal structures originates (possibly the amygdala or hippocampus). When the function of the GPh is taken over by other extrapyramidal circuits, however, the input would come from the human striatum, and the output from the pallidum would be GABAergic. Moreover, the effect of nicotinergic cholinergic agents should be studied because the output of the medial habenula to the interpenduncular nucleus is substantially cholinergic (Viswanath et al., 2014; Antolin-Fontes et al., 2015).

### Conflict of interest statement

AJML received a speaker's fee and an unconditional research grant from Servier Pharma Netherlands. SAI has nothing to disclose. The authors declare that the research was conducted in the absence of any commercial or financial relationships that could be construed as a potential conflict of interest.

## Footnotes

^1^http://en.wikipedia.org/wiki/, viewed on November 20, 2014.
